# Emphatic information on bone mineral loss using quantitative ultrasound sonometer for expeditious prediction of osteoporosis

**DOI:** 10.1038/s41598-023-44407-w

**Published:** 2023-11-08

**Authors:** Kottaimalai Ramaraj, Pallikonda Rajasekaran Murugan, Gautam Amiya, Vishnuvarthanan Govindaraj, Muneeswaran Vasudevan, M. Thirumurugan, Yudong Zhang, S. Sheik Abdullah, Arunprasath Thiyagarajan

**Affiliations:** 1 Department of Electronics and Communication Engineering, Kalasalingam Academy of Research and Education, Krishnankoil, Tamilnadu India; 2 Department of Computer Science and Engineering, Kalasalingam Academy of Research and Education, Krishnankoil, Tamilnadu India; 3 Data Science Division, School of Computing Science and Engineering, VIT-Bhopal, Sehore, Madhya Pradesh India; 4 MGR Medical University, Chennai, Tamilnadu India; 5 School of Computing and Mathematical Sciences, University of Leicester, Leicester, LE1 7RH UK

**Keywords:** Osteoarthritis, Clinical trials

## Abstract

Osteoporosis (OP) and osteoarthritis (OA) are skeletal disorders characterized by a reduction in bone density and quality, resulting in increased fragility and susceptibility to fractures. These illnesses are exhibiting a higher prevalence among both males and females. Fracture risk is determined by using the BMD score (Bone Mineral Density). Looking at the bone loss that comes with osteoarthritis (OA) and osteoporosis (OP), this study also looks at the technological methods used to test for these conditions in order to improve therapies and treatment plans for older people. As a matter of consideration, the prevalence of osteoporosis is higher among postmenopausal women (20%) compared to premenopausal women (14.28%) and males (6.77%). The utilization of a preliminary calcaneal Quantitative Ultrasound (QUS) examination is warranted in order to effectively handle the matter of osteoporosis. The prompt assessment of a patient can provide valuable insights into potential fractures and aid in the prevention of bone injury. In a nutshell, it is imperative to comprehend the impact of OA (osteoarthritis) and OP (osteoporosis) on bone health in order to effectively manage the escalating apprehensions surrounding these conditions. Sophisticated diagnostic techniques, such as the calcaneal quantitative ultrasound (QUS) test, have the potential to enhance the well-being of older individuals by enabling early detection and treatment of many ailments.

## Introduction

Bones, being living tissues, provide essential functions in terms of offering structural support to the human body, enabling locomotion, and protecting internal organs^1^. Osteoporosis, a medical disorder defined by the progressive loss of bone density and increased susceptibility to fractures, presents a substantial health issue^1^. The term "osteoporosis" is derived from the Greek words osteo, meaning bone, and porosis, meaning porous. This condition is characterized by a reduction in bone density, leading to an increased risk of fractures. The consequences of osteoporosis encompass not only physical discomfort resulting from fractures but also functional limits and difficulties in carrying out routine activities^2^.

**Figure 1:**
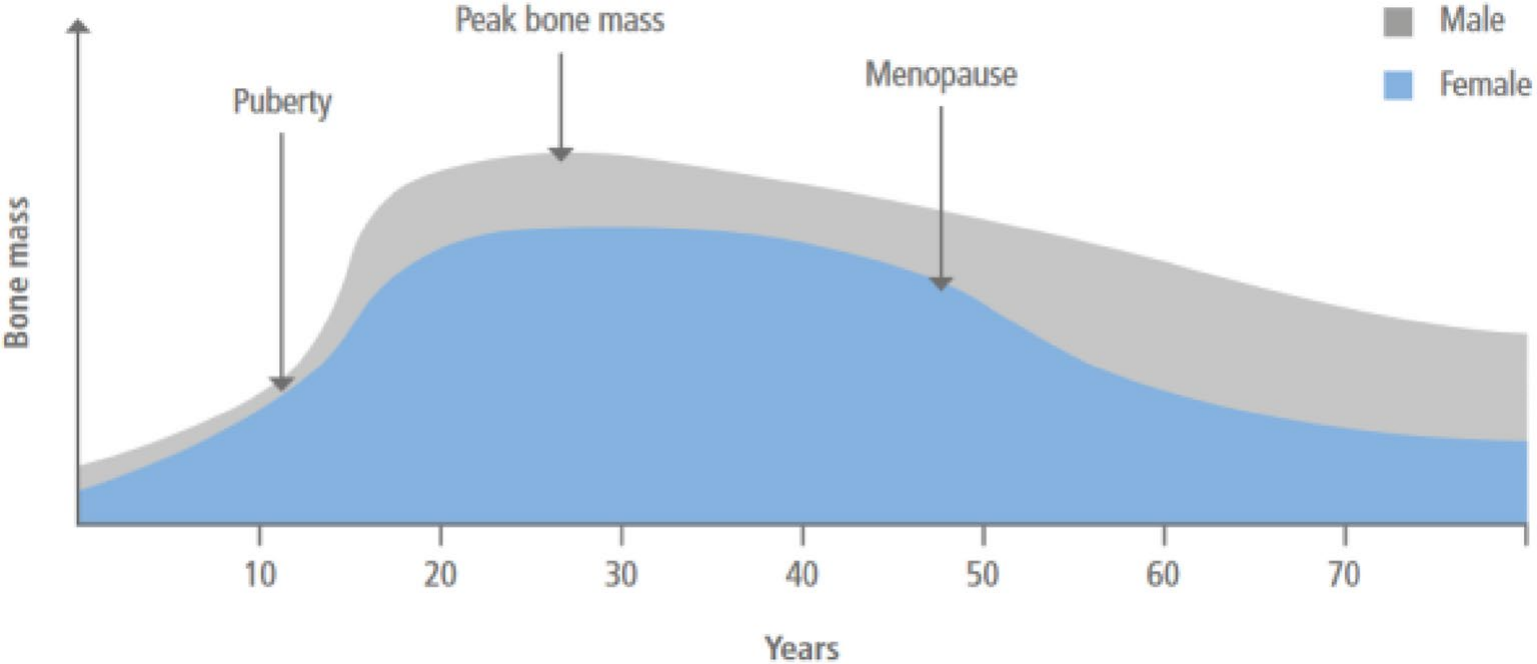
Temporal dynamics of bone mass variation across the lifespan*.

Fractures in the spine, wrist, and hip regions are a frequent consequence of osteoporosis^3^. On a global scale, an estimated 1.6 million hip fractures transpire each year, and it is anticipated that this figure will escalate to 6.3 million by the year 2050. This surge is particularly likely to be prominent in regions beyond the United States and Europe^4^. It is projected that by the year 2050, Asia will be responsible for more than 50% of all hip fractures associated with osteoporosis^5^. Figure 1 depicts the notable decrease in bone mass subsequent to menopause, typically occurring at approximately 50 years of age, which corresponds to the decline in estrogen levels^6^. Fortunately, several therapeutic interventions have exhibited the capacity to mitigate the likelihood of hip fractures by as much as 40%, vertebral fractures by 30–70%, and non-vertebral fractures by 15–20%^7^.

The screening of bone mineral density (BMD) provides a non-invasive and secure method for the diagnosis of osteoporosis and the evaluation of an individual's likelihood of having the condition in the future^8^. Standard X-rays, which are mostly used to find broken bones, cannot reliably measure bone mineral density (BMD)^9^. Because of this, professional imaging methods are needed to measure BMD. There are various screening methods for bone mineral density (BMD), such as Dual Energy X-ray Absorptiometry (DEXA/DXA), Magnetic Resonance Imaging (MRI), and Quantitative Computed Tomography (QCT). Nevertheless, the technology that has gained the most widespread adoption and is highly recommended is DXA imaging^10^. The assessment and management of health concerns connected to osteoporosis heavily rely on the measurement of bone mineral density (BMD) due to its significant correlation with the likelihood of experiencing fractures in the future^11^.

In the preceding 30 years, a multitude of safe, precise, and dependable methodologies for quantifying bone density have been devised^12^. The majority of these techniques entail the utilization of ionizing radiation, specifically X-rays, and are dependent on the reduction of energy beams as they traverse soft tissues and bone^13^.

One novel methodology entails the utilization of acoustic energy, specifically ultrasound waves, for the purpose of evaluating bone integrity and forecasting the likelihood of fractures^14^. The interaction between ultrasound, which is a type of sound wave, and bone differs from that of ionizing electromagnetic radiation. This distinction provides distinct and valuable information regarding bone properties^15^. The utilization of Quantitative Ultrasound (QUS) technology has emerged as a viable alternative approach for the assessment of Bone Mineral Density (BMD). Quantitative ultrasound (QUS) is increasingly becoming recognized as a valuable tool for evaluating bone health, mostly due to its notable advantages in terms of efficiency, cost-effectiveness, and lack of radiation exposure when compared to conventional techniques such as computed tomography (CT), magnetic resonance imaging (MRI), and dual-energy X-ray absorptiometry (DEXA/DXA)^16^.

The evaluation of two important variables, Broadband Ultrasound Attenuation (BUA) and Speed of Sound (SoS), can be conducted using ultrasound technology as it passes through both bone and soft tissues^17^. These criteria offer a full assessment of bone density, strength, and the potential for future fractures. The speed of sound (SoS) is quantified in millimeters per second, denoting the duration necessary for ultrasound waves to traverse a particular distance within the calcaneus bone^18^. The method known as BUA, or broadband ultrasonic attenuation, quantifies the changes in ultrasound attenuation as a function of the frequency of the sound wave being emitted. This measurement is typically denoted in decibels per megahertz (dB/MHz). The stiffness index, often referred to as the rigidity index or QUS index, is commonly expressed as a percentage relative to the values observed in young adults or as a percentage relative to weight-matched reference values, as specified by the manufacturer. The calcaneus bone is commonly chosen as the primary location for quantitative ultrasound (QUS) assessment due to its primarily cancellous bone composition, inclusion of soft tissues, and provision of a spacious and flat surface that facilitates straightforward evaluation^19^.

A few recent studies have emphasized how useful it is to use quantitative ultrasound (QUS) testing at the calcaneal bone to find osteoporosis (OP) early on. This methodology holds significant value due to its radiation-free nature, small design, and uncomplicated implementation, rendering it highly ideal for extensive utilization in clinical settings.

In addition to osteoporosis, it's worth noting that osteoarthritis (OA), another common musculoskeletal disorder, can also affect bone health and mobility. OA involves the degeneration of joint cartilage and can lead to pain and functional limitations. Assessing both OP and OA is crucial for a comprehensive musculoskeletal health evaluation, which could also be accomplished using the QUS^18^.

## Results

**Figure 2:**
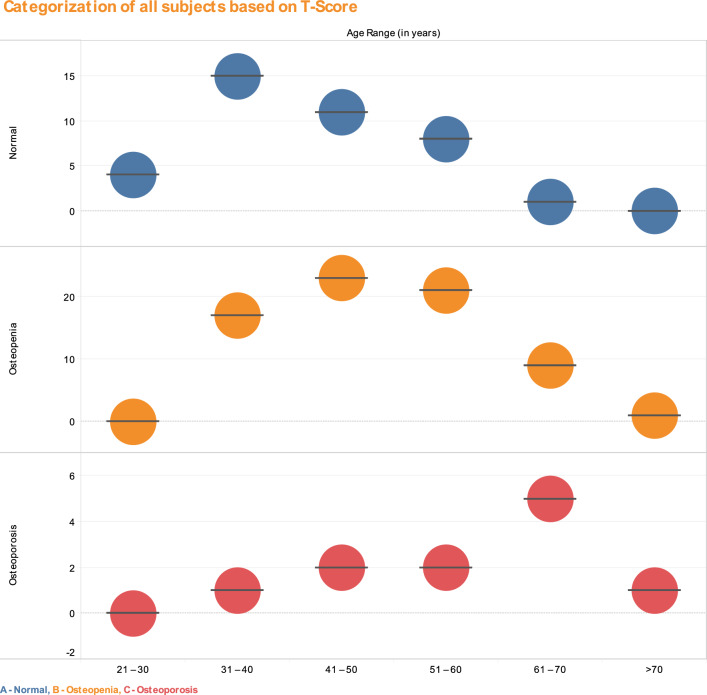
Categorization of both genders (all subjects) based on T-score at different ages.

Of the 121 subjects, including young people and elders, 39 cases (32.23%) had normal or typical bone mass, 71 cases (58.67%) had decreased bone mass, namely osteopenia, and 11 cases (9.09%) had osteoporosis. The significant proportion of normal bone mass and bone mass loss drastically diminished with age. Figure 2 describes the number of subjects in different age ranges, which are categorized as normal, Osteopenic, and Osteoporotic based on T-score values attained from the QUS based BMD test.

**Figure 3:**
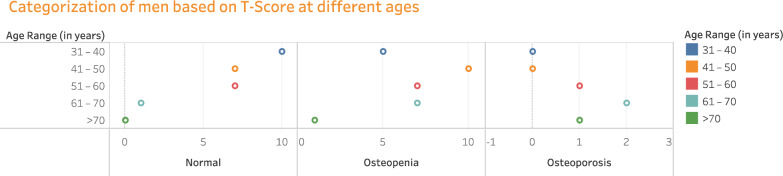
Classifying the disease occurrence in men of various age groups based on T-scores.

The number of men (59 in headcount) in different age groups is categorized as normal, Osteopenic, and Osteoporotic based on T-score values attained from the QUS based BMD test and is illustrated in Fig. 3. Out of 59 men from overall subjects, 25 men (42.37%) had normal bone mass, 30 men (50.84%) had reduced bone mass/osteopenic, and 4 men (6.77%) had severe bone mass loss/osteoporotic.

**Figure 4:**
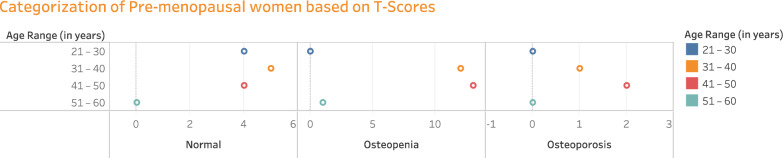
Classifying the disease occurrence in premenopausal women of various age groups based on T-scores.

The premenopausal women (42 in headcount) in different age groups are categorized as normal, Osteopenic, and Osteoporotic based on T-score values achieved from the QUS-BMD test, as exemplified in Fig. 4. Among the 121 participants, 62 are women. Again, the female participants are categorized as premenopausal (42 cases/67.74%) and postmenopausal (20 cases/32.25%) women. Out of 42 premenopausal women from overall subjects, 13 subjects (30.95%) had normal bone mass, 26 subjects (61.9%) had reduced bone mass/osteopenic, and 3 subjects (14.28%) had severe bone mass loss/osteoporotic.

**Figure 5:**
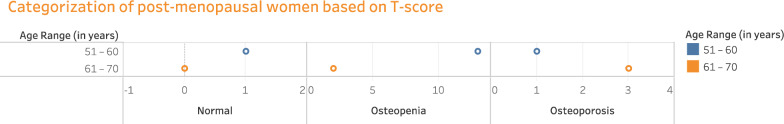
Classifying the disease occurrence in postmenopausal women of various age groups based on T-scores.

The postmenopausal women (20 in headcount) in different age groups are categorized as normal, Osteopenic, and Osteoporotic using T-score values achieved from the QUS based BMD test, as represented in Fig. 5. Out of 20 postmenopausal women from overall subjects, 1 subject (5%) had normal bone mass, 15 subjects (75%) had reduced bone mass/osteopenia, and 4 subjects (20%) had severe bone mass loss/osteoporotic.

The overall subjects (121 in headcount) are grouped as premenopausal women, postmenopausal women, and men. The assessment of normal, osteopenic, and osteoporotic bone mass is accomplished using the T-score attained from the BMD test and is consolidated in Table 1.

**Table 1:** Assessment of osteoporosis in all subjects based on T-score.

Group	Number of subjects	Normal (n, %)	Osteopenia (n, %)	Osteoporosis (n, %)
Premenopausal women	42	13 (30.95)	26 (61.9)	3 (14.28)
Postmenopausal women	20	1 (5)	15 (75)	4 (20)
Men	59	25 (42.37)	30 (50.84)	4 (6.77)

**Figure 6:**
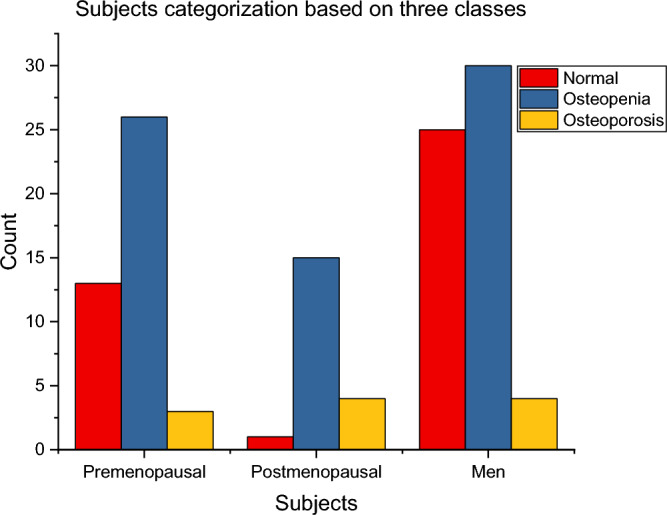
Categorization of subjects as per the three cases.

The findings indicate that a higher percentage of osteoporosis cases is reported in postmenopausal women (20%) than in premenopausal women (14.28%) and men (6.77%). The categorized subjects as per the three cases are depicted in Fig. 6.

According to the findings from this evaluation, it is observed that 4 postmenopausal women, 3 premenopausal women, and 4 men have suffered from osteoporosis, and they will have a higher possibility of bone fracture or damage. Likewise, 26 postmenopausal women, 15 premenopausal women, and 30 men have suffered from osteopenia. The remaining 13 postmenopausal women, 1 premenopausal woman, and 25 men are found to have good bone health. All these results have been derived using the T-score assessment of participants.

The clinician associated with this research during the data collection has acknowledged/certified/authorised/acceded the outcomes from this analysis. Furthermore, the necessary recommendations to the participants to enhance their bone strength and preventive measures and precautions to be followed/adopted/obliged to suppress bone fractures/damages have been adequately provided by the clinician based on the diagnostic results.

**Figure 7:**
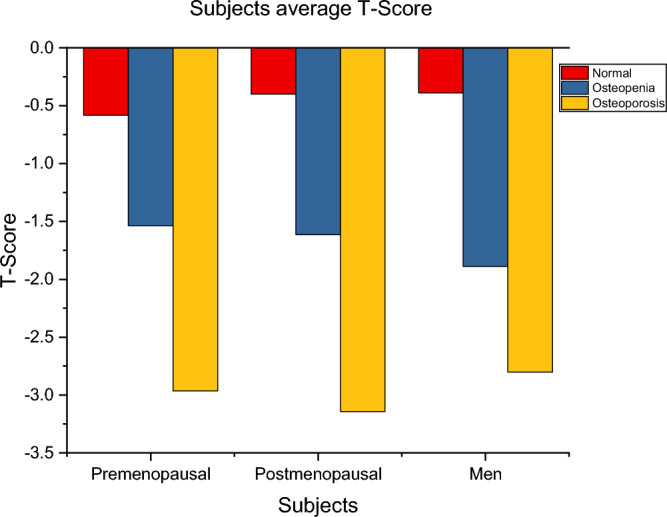
Average T-score of different subjects.

Figure 7 portrays the average T-score value for men and pre- and postmenopausal women in normal, osteopenic, and osteoporotic conditions. The average T-score in osteoporotic postmenopausal women is discovered to be exceptionally low when contrasted to men and premenopausal women, and they are at a higher risk of developing osteoporosis breakage^20^.

## Discussion

The BMD data collected from 121 participants was categorized and analysed based on the T-score values obtained upon diagnosing them. From that analysis, the above-mentioned results were obtained.

Out of 121 patients, for some special or specific reasons including weight, obesity, height, and leg bone structure, the BMD test was conducted on 14 subjects in four ways: testing both legs one by one while in a sitting or standing position. Following are some of the interesting findings attained from the test outcomes.

- The T-score of 10 subjects obtained using the BMD test from the left leg sitting position indicates the presence of osteopenia in their bones. The average weight of the subjects is 56.5 kg. The T-scores analysed for the left leg while standing and the right leg in both sitting and standing positions indicate the presence of osteopenia exactly.
- The T-score of the two subjects tested in the left leg sitting position indicates normal bone mass. Also, the T-score of the same subjects represented normal bone mass while tested on the left leg in a standing position. The results demonstrate the presence of normal bone mass in those subjects.
- The T-score of a subject tested in the left leg sitting position indicates normal bone mass. Also, the T-score of the same subject reveals normal bone mass while tested on the right leg in the standing position. The results demonstrate the presence of normal bone mass in that subject.
- The T-score of a subject tested in the left leg sitting position indicates the presence of osteoporosis. Also, the T-score of the same subject represents osteoporosis while tested on the left leg during standing posture. The results demonstrate the presence of extremely reduced bone mass in that subject.

## Materials and methods

### Participant details

The experiment has been carried out at the Kalasalingam Academy of Research and Education, Krishnankoil, and Poovani village located near Srivilliputtur, Tamilnadu, India. All the subjects provided informed consent, and the investigation was accepted by the local ethics committee. The information was collected under the supervision of a healthcare professional. This study involves 121 participants, ranging in age from 21 to 87 years old. Out of 121 participants, 59 are men between the ages of 32 and 87, 42 are premenopausal women between the ages of 21 and 51, and 20 are postmenopausal women between the ages of 52 and 70.

The acquisition of the BMD values from the participants was well consented to after duly explaining the procedure, which is purely intended for the purpose of the study and not for any other personal benefits. The entire sequence of actions involved were apparently approved by the ethical committee constituted by the Institutional Research Board of Kalasalingam Academy of Research and Education, Krishnankoil, Tamilnadu, vide Reference Number: KARE/CEC/MOM/2022-23/01 dated on 27.03.2023 (KARE-Kalasalingam Academy of Research and Education; CEC—Code of Ethics Committee; MOM—Minutes of Meeting). All the ethical standards and practices were strictly adhered to during the BMD data acquisition from live human subjects, and the same has been available for perusal in the supporting file information.

The baseline characteristics of three different groups are displayed in Table 2: men and pre- and postmenopausal women. The basic characteristics include subjects’ age, height, weight, body muscle index (BMI), and menopause information for women.

**Table 2:** Baseline characteristics collected through the medical camp organized.

Characteristics	Premenopausal women	Postmenopausal women	Men
Number of subjects	42	20	59
Age (years)	40.2	58.5	50.16
Height (cm)	154.06	151.96	166.17
Weight (kg)	58.19	56.46	67.61
BMI (kg/m^2^)	24.44	24.26	24.26
Age at menopause	–	49.5	–
Years since menopause	–	7.55	–

### Equipment details

The BMD score of the participants is acquired using Ultrasound Bone Densitometer of Model number/name CM-200 light, manufactured by Furuno Electric Co., Ltd., Japan. The device requires a supply voltage of 200–240 V AC, a frequency of 50/60 Hz, and a maximum current of 0.3A. It belongs to a Class 1 Type B medical device. The functional keys and colour LCD give readers easy-to-follow instructions. An embedded printer prints the outcome in graph representation on a chart. Using the ultrasound pulse penetration technique, the Speed of Sound (SOS), and T-score are measured at the Heel bone (Calcaneus) site. The US produces a 500 kHz center frequency. The operating conditions include a temperature of 10–35 °C and a humidity of 35–85% RH. The device is 495 mm (width) × 310 mm (depth) × 200 mm (height) and weighs about 9 kg.

The footplate has five levels that can be adjusted in order to precisely determine the heel bone's centre. Temperature has a tendency to affect measuring results. Utilizing a temperature sensor to adjust for temperature, the CM-200 light offers extremely accurate readings. With the CM-200, specialized application software for data administration is offered. The application programme that comes with the CM-200 light's interface to a PC allows for data administration as well as accuracy monitoring and patient data enrollment. Bluetooth enables wireless connectivity between the device and the computer. It maximises the amount of space available for installation or operating agility and can be devoid of tedious wiring. Software used for CM-200 light data management is offered as a standard accessory and is simple to use, taking about 10 s for the assessment.

### Data collection and measurement method

Ultrasound measurements of bone strength have been identified as a challenging approach for assessing the risk of osteoporotic fracture and revealing additional data regarding the bone structure and composition. The defined approach based on ultrasound for measuring the speed of sound (SoS) and broadband ultrasound attenuation (BUA) at the Calcaneum site helps in assessing the variations in low bone mass.

For the detection and evaluation of osteoporosis, a quantitative ultrasound (QUS) Bone Sonometer (BS) is employed to analyse bone status by assessing the stiffness index in the heel (calcaneus bone). Measures are taken with the participant seated and the left foot kept on the foot positioner^18^. The following data were collected for some special cases: measuring BMD in both legs while participants are asked to sit first and then stand.

During BMD quantification, the heel clamp assists the participant in keeping the foot stable and the heel aligned with the transducers. The Calcaneus bone was preferred as a monitoring site due to it being an overstressed, weight-bearing bone that is extremely productive in the remodeling process, revealing improvements within bone tissue sooner than compact bone^14^.

Because there is minimal soft tissue encompassing the Calcaneus bone, it is an ideal location for assessment, thus determining a patient's fracture likelihood. The electrical pulse is converted to a sound wave by the transmitter presented on one side of the heel. It travels through soft tissue and Calcaneus bone before arriving at the receiver as a sound wave. The obtained sound wave is transferred as an electrical signal, which the QUS-BS analyses using the default program. QUS-BS assesses the SoS and BUA and calculates the stiffness index. The T-score and Z-score expressed by the stiffness index results are employed to assist clinicians in the determination of osteoporosis.

$$\mathrm{SI}=\left[ \left(0.67*\mathrm{BUA}\right)+\left(0.28*\mathrm{SOS}\right)\right]-420$$

Approximately, the experiment took about two to three minutes to assess BMD for a participant. The procedure during the experiment includes: cleaning the foot of the participant; cleaning the foot plate of the equipment; applying the gel to both the transmitter and receiver sections to have good contact over the skin; ensuring the patient’s leg is placed at the right angle (the leg should be straight and not inclined/elevated/deviated from the US signal course); passing ultrasound waves to the Calcaneal bone upon pressing the start button; and calculating the T-score value and their corresponding remarks as a result. On the whole, the duration required for the entire acquisition of data from the volunteers/participants/needy was for a whole day. All these have been vivaciously done to assimilate the data, not only for the purpose of research but also to provide wide and sufficient awareness and sensitize the public regarding the enigmatic and adverse effects of OA and OP.

### T-score and Z-score

The T-score is the most important parameter for assessing osteoporosis because it compares the subject's BMD to the average BMD of a normal young adult. T-scores greater than − 1 are considered normal; T-scores between − 1 and − 2.5 are considered osteopenic (the initial stage of osteoporosis); and T-scores less than − 2.5 are considered osteoporotic, indicating the risk for fractures.

The Z-score compares the subject's BMD to the average BMD of a person of the same age. A Z-score of less than − 2 indicates that the patient has less bone mass and/or is losing bone. The above correlation defines whether a subject differs significantly from the expected pattern for his or her age and gender^21^. Table 3 depicts the Standard Deviation (SD) and T-score values based on bone mass variation.

**Table 3:** SD and T-score values based on bone mass variation.

Diagnostic category	Standard deviation value (SD)	T-score
Healthy bone mass	BMD within 1 SD of the reference mean for young adults	T-score > − 1
Lone bone mass/osteopenia	BMD between 1.0 and 2.5 SD below the mean for young adults	T-score between − 1 and − 2.5
Osteoporosis	BMD of ≥ 2.5 SD below the mean for young adults	T-score < − 2.5
Severe or established osteoporosis	BMD of ≥ 2.5 SD below the mean for young adults in the presence of one or more fractures	T-score < − 2.5

### Informed consent

Informed consent was obtained from all individual participants included in the study.

## Summarization of the contributions made in the paper

The research presented in this paper addresses the critical issue of osteoporosis, a condition characterized by reduced bone mass and increased fracture risk. Osteoporosis is a major health concern worldwide, particularly affecting postmenopausal women. As a promising and non-invasive way to check bone health and predict osteoporosis, this paper looks into the use of quantitative ultrasound sonometer (QUS) technology on the calcaneus bone.

Key findings and contributions of the research include:

1. Prevalence of osteoporosis: the study reveals that postmenopausal women have a significantly higher percentage of osteoporosis cases (20%) compared to premenopausal women (14.28%) and men (6.77%). This highlights the importance of early detection and management, particularly in postmenopausal women, who are at greater risk of fractures.
2. QUS as a diagnostic tool: the research emphasizes the effectiveness of calcaneal QUS as a preliminary screening method for osteoporosis. It is faster, less expensive, and non-radiation compared to conventional methods like Dual Energy X-ray Absorptiometry (DEXA/DXA). This makes it a practical option for widespread clinical use.
3. T-score and Z-score analysis: the paper discusses the use of T-scores and Z-scores to assess bone mineral density. T-scores are compared to the average BMD of a normal young adult, while Z-scores consider the patient's age. These scores help identify individuals at risk for osteoporosis and bone loss.
4. Individualized assessment: the research also presents cases where the BMD test was conducted on specific subjects in different positions and leg orientations. This approach provides insights into the variations in bone mass among individuals, taking into account factors like weight, obesity, and leg bone structure.
5. Clinical implications: the study not only focuses on diagnosis but also emphasizes the importance of clinicians providing recommendations and preventive measures based on diagnostic results. This holistic approach to patient care can help reduce the risk of fractures and bone damage.

In summary, this research underscores the significance of early detection and management of osteoporosis, especially in postmenopausal women, using quantitative ultrasound sonometer technology. The use of T-scores and Z-scores allows for individualized assessments, and the study emphasizes the role of healthcare professionals in providing guidance to patients based on diagnostic results.

## Conclusion

It has been determined that quantitative ultrasound (QUS) can be effectively employed for the analysis of bone mineral loss. Furthermore, QUS demonstrates clear differentiation between those with normal bone density and those with osteoporosis, thereby establishing itself as a valuable tool in the diagnosis and treatment of osteoporosis. This study assessed the efficacy of calcaneal quantitative ultrasound (QUS) as a diagnostic tool for osteoporosis as well as its potential for screening large populations in a more efficient and cost-effective manner. Consequently, it is recommended that calcaneal quantitative ultrasound (QUS) be considered as an initial screening tool for those with a pre-existing risk of osteoporosis, as well as those with intermediate osteoporosis, in order to reduce the risk of fractures. Based on empirical evidence, it has been determined that osteoporosis has a higher incidence rate among women in the postmenopausal stage, hence rendering them more susceptible to fractures compared to premenopausal women and men. Based on the results of this investigation, it has been observed that postmenopausal females have a diminished T-score value in comparison to both premenopausal females and males. The rate of decline in bone mineral composition is more rapid during the first 5 years following menopause, but it gradually slows down as individuals age. Moreover, in order to mitigate the possibility of misinterpretation and unsuccessful diagnosis, the evaluation and diagnosis of atypical bone density could potentially incorporate an assessment of the patient's clinical symptoms, biochemical markers, diverse bone metabolic characteristics, and other pivotal aspects. One limitation of this study is the relatively small sample size, which necessitates the expansion of the investigation in order to facilitate a more comprehensive analysis. The investigation serves to ascertain the variability of bone mass across three distinct classes of people spanning various age ranges. Physicians are able to make timely diagnoses and implement treatment strategies for patients by utilizing their T-score results.

## Data Availability

The datasets generated during and/or analysed during the current study are available from the corresponding author on reasonable request.
